# SKAP, an outer kinetochore protein, is required for mouse germ cell development

**DOI:** 10.1530/REP-15-0451

**Published:** 2016-03

**Authors:** Corinne Grey, Julien Espeut, Rachel Ametsitsi, Rajeev Kumar, Malgorzata Luksza, Christine Brun, Marie-Hélene Verlhac, José Angél Suja, Bernard de Massy

**Affiliations:** 1 CNRS, IGH (UPR1142), 141 rue de la Cardonille, 34396, Montpellier, France; 2 CNRS, CRBM (UMR5237), 1919 route de Mende, 34293 Montpellier, France; 3 INRA, Département Biologie et Amélioration des Plantesroute de Saint-Cyr, 78026 Versailles, France; 4 Collège de France, CIRB (UMR CNRS 7241/INSERM-U1050)Place Marcelin Berthelot, 75005 Paris, France; 5 Departamento de Biología, Facultad de Ciencias, Universidad Autónoma de Madrid, 28049 Madrid, Spain

## Abstract

In sexually reproducing organisms, accurate gametogenesis is crucial for the transmission of genetic material from one generation to the next. This requires the faithful segregation of chromosomes during mitotic and meiotic divisions. One of the main players in this process is the kinetochore, a large multi-protein complex that forms at the interface of centromeres and microtubules. Here, we analyzed the expression profile and function of small kinetochore-associated protein (SKAP) in the mouse. We found that two distinct SKAP isoforms are specifically expressed in the germline: a smaller isoform, which is detected in spermatogonia and spermatocytes and localized in the outer mitotic and meiotic kinetochores from metaphase to telophase, and a larger isoform, which is expressed in the cytoplasm of elongating spermatids. We generated SKAP-deficient mice and found that testis size and sperm production were severely reduced in mutant males. This phenotype was partially caused by defects during spermatogonia proliferation before entry into meiosis. We conclude that mouse SKAP, while being dispensable for somatic cell divisions, has an important role in the successful outcome of male gametogenesis. In germ cells, analogous to what has been suggested in studies using immortalized cells, SKAP most likely stabilizes the interaction between kinetochores and microtubules, where it might be needed as an extra safeguard to ensure the correct segregation of mitotic and meiotic chromosomes.

## Introduction

Mammalian gametogenesis is a complex and highly orchestrated process during which germ cells differentiate into mature haploid gametes. At birth, seminiferous cords of mammalian testes contain germ cells that are arrested in the G0 phase of the cell cycle. In rodents, these germ cells resume mitosis shortly after birth and migrate to the basement membrane of the seminiferous tubules where they begin to differentiate into pluripotent type A spermatogonia, which are capable of self-renewal or entering spermatogenesis (McGuinness & Orth 1992, de Rooij & Grootegoed 1998). Type A spermatogonia that enter spermatogenesis differentiate progressively into type B spermatogonia while undergoing several rounds of mitotic cycles. In turn, type B spermatogonia enter meiotic S-phase and give rise to pre-leptotene spermatocytes (de Rooij & Russell 2000). In mouse, spermatocytes enter meiosis around 8 days after birth and spend 12 days in prophase I before reaching metaphase I. Concomitantly with their progression through prophase I, spermatocytes physically move from the basal lamina of the seminiferous tubules towards the lumen. After diakinesis, they rapidly undergo meiosis II, which is similar to somatic mitosis, and whose products give rise to haploid round spermatids. Finally, during spermiogenesis, spermatids progressively differentiate to become functional spermatozoa. Whereas in males meiosis takes place after birth, in females the first steps of meiosis take place during embryogenesis. At birth, oocytes are embedded within primordial follicles and arrested at the dictyate stage, the last step of meiotic prophase I. Upon ovulation, oocytes progress through metaphases I and II where they are arrested again. Fertilization then triggers resumption of meiosis II.

Proper gametogenesis relies on dynamic changes of the cytoskeleton, organelle movements and particularly on accurate chromosome segregation. During gametogenesis, both mitotic and meiotic chromosome segregation are dependent on the faithful organization of kinetochores, which are large multi-protein complexes that form at the surface of the centromeres where they mediate the attachment of chromosomes to spindle microtubules. If not correctly attached, the kinetochores generate a signal that activates the spindle assembly checkpoint (SAC) to prevent entry into anaphase and completion of division (Musacchio & Salmon 2007).

Here, we describe the role of mouse small kinetochore-associated protein (SKAP) during gametogenesis. SKAP was first described as a G2 phase-induced gene that is upregulated in numerous human tumors (Fang *et al*. 2009). In HeLa and DT40 cells, SKAP localizes at spindle poles during mitosis and at the outer kinetochores from metaphase to telophase (Fang *et al*. 2009, Schmidt *et al*. 2010). Human SKAP (hSKAP) was also named kinastrin, as it forms a complex with astrin, a protein recruited to microtubule plus ends, spindle poles (Dunsch *et al*. 2011) and aligned kinetochores (Schmidt *et al*. 2010). The localization of SKAP at the kinetochores depends on the direct interaction with Mis13, which is part of the KMN (Knl-1/Mis12 complex/Ndc80 complex) core microtubule binding site, located at the outer kinetochores (Wang *et al*. 2012). In immortalized cells, downregulation of SKAP by siRNA leads to a mitotic delay with several mitotic defects such as the formation of multipolar spindles and chromosome misalignments (Fang *et al*. 2009, Schmidt *et al*. 2010, Dunsch *et al*. 2011). In HeLa cells, SKAP also interacts with the kinesin CENPE, known to orchestrate faithful kinetochore–microtubule interaction (Yao *et al*. 2000, Huang *et al*. 2012). However, there are no studies on SKAP function during mammalian meiotic divisions.

In this work, we found that SKAP expression is germline-specific. The protein localizes at the outer kinetochores of metaphase to anaphase spermatogonia and in spermatocytes during both meiotic divisions. Knockout of *Knstrn*, the gene encoding SKAP, resulted in smaller testes and a strong decrease in sperm production compared with WT littermates. In most seminiferous tubules of SKAP-deficient testes, germ cells were partly or completely absent, even before the onset of meiosis. We suggest that SKAP has an important role in spermatogonia proliferation to ensure the production of a sufficient amount of germ cells that are able to enter meiosis and produce enough spermatozoa for normal fertility.

## Materials and methods

### SKAP-deficient mice (Knstrn^tm1d(KOMP)Wtsi^)

ES clones that contain a conditional gene-target cassette upstream of the third exon of *Knstrn* (MGI: 4362164, ES cell clone: EPD0125_3_H05, cell line JM8.N4, purchased from EUCOMM) were produced. Briefly, the L1L2_Bact_P cassette was inserted at position 118817419 of chromosome 2 upstream of exon 3 (Build GRCm38). The original cassette was composed of an FRT site followed by the *lacZ* sequence and a *loxP* site. This first *loxP* site was followed by the neomycin resistance gene under the control of the human beta-actin promoter, by the SV40 polyA, a second FRT site and a second *loxP* site. A third *loxP* site was inserted downstream of exon 3 at position 118818211. Exon 3 was thus flanked by *loxP* sites. The ‘conditional ready’ allele Knstrn^tm1d(KOMP)Wtsi^ was created by FLP recombinase expression in mice carrying this allele and subsequent CMV-Cre expression resulting in knockout mice lacking exon 3 of the *Knstrn* gene. All mouse experiments were carried out according to the CNRS guidelines with the approval of the Languedoc-Roussillon Animal Care and Use Committee (CEEA-LR-11028).

### Northern blotting and RT-PCR

Total RNA from various mouse tissues was prepared with the Gene Elute Mammalian Total RNA purification kit (Sigma), according to the manufacturer's instructions. After electrophoresis of 10 μg of total RNA of each tissue and transfer to nitrocellulose membranes (GE Healthcare), northern blot analysis was performed with an α-32P-dCTP-labeled probe specific for exons 2–7 of *Knstrn* (obtained using *Knstrn*-specific oligonucleotides: mORF236U19 (5′-TGG CCA GTG CTA AGA CGG T-3′) mORF681L19 (5′-CTT TGA TGC CAG GGT CTC T-3′).

For RT-PCR amplification, first-strand DNA was synthesized by using oligo d(T)18, SuperScriptIII (Invitrogen) and total RNA (1–2 μg) extracted from various tissues and from testes of juvenile mice. cDNA was PCR-amplified with the following *Knstrn*-specific primer pairs: mORF236U19 (5′-TGGCCAGTGCTAAGACGGT-3′) and mORF681L19 (5′-CTTTGATGCCAGGGT CTCT-3′). PCR cycling conditions were as follows: 3 min at 94 °C, and then 35 cycles of 15 s at 94 °C, 30 s at 56 °C, 30 s at 72 °C, and 5 min at 72 °C. Primers to amplify actin as a control were as follows: forward 5′-GCCGGCTTACACTGCGCTTCTT-3′ and reverse 5′-TTCTGGCCCATGCCCACCAT-3′. PCR cycling conditions were as follows: 3 min at 94 °C, and 35 cycles of 15 s at 94 °C, 30 s at 59 °C, 30 s at 72 °C and 5 min at 72 °C.

### Antibodies

Antibodies against full-length SKAP were raised by immunizing two guinea pigs with full-length recombinant SKAP protein fused to a 6× HIS tag at the N-terminal (Covalab, Villurbaine, France). SKAP polyclonal serum was then affinity-purified by incubation with a nitrocellulose membrane containing immobilized SKAP recombinant protein. The N-terminal specific anti-SKAP antibody was raised against a peptide covering amino acids 1 to 78 in I.M. Cheeseman's laboratory (Whitehead Institute for Biomedical Research, Cambridge, MA, USA). We also used home-made guinea pig anti-SYCP3 as in Grey *et al*. (2009) and mouse monoclonal anti-SYCP3 (Abcam, ab-12452), mouse monoclonal anti-phospho-Histone H2A.X (Ser139) (Upstate-Millipore 05-636), human ACA/CREST serum (Europa Bioproducts, Cambridge, UK, FZ90C-CS1058), rat anti-tubulin (YOL1/34, Abcam, ab-6161), rabbit anti-GFP (Torrey Pines-401, Clinisciences, Nanterre, France), mouse anti-tubulin clone DM1a (Sigma T6199), rat anti-TRA98 (Abcam, ab-82527) and rabbit anti-SOX9 antibody, kindly provided by B Boizet's laboratory (De Santa Barbara *et al*. 1998). Secondary antibodies were as follows: goat anti-guinea pig Alexa Fluor 488 (Life Technologies, A-11073, 1:300); goat anti-rabbit Alexa Fluor 555 (Life Technologies, A-21428, 1:200); goat anti-rat Alexa Fluor 488 (Life Technologies, A-21212, 1:800); donkey anti-mouse Alexa Fluor 647 (Life Technologies, A-31571, 1:200); donkey anti-guinea pig IgG HRP-conjugated (Jackson ImmunoResearch Laboratories, Newmarket, Suffolk, UK, 706-035-148); and donkey anti-rabbit IgG HRP-conjugated (Jackson ImmunoResearch Laboratories, 711-035-152).

### Plasmids

Full-length cDNA was amplified from testes and sub-cloned into different vectors (pET15, pDest15 and pDest27) using the Gateway technology (Life Technologies) with the following primer pairs: SKAPattU (5′-GGGGACAAGTTTGTACAAAAAAGCAGGCTTCGCGGCTCCCGAGGCCGA-3′) and SKAPattL (5′-GGGGACCACTTTGTACAAGAAAGCTGGGTACTACATTTCTAAGAGCTGCTCCA-3′).

### SKAP extraction and western blotting

For western blotting experiments, testes were decapsulated and homogenized in high salt buffer (1% Triton X-100, 400 mM NaCl, 50 mM HEPES-Na pH 7.4, 4 mM DTT, 1× EDTA-free complete protease inhibitor; Roche). Lysates were then centrifuged in a microfuge at 4 °C at 21 000***g*** for 10 min and pellets were discarded. For western blotting, extracts (50 μg/each) were separated on 7.5% TBX-acrylamide gels (Bio-Rad) and then blotted on nitrocellulose membranes, blocked and incubated overnight at 4 °C with the anti-SKAP (1:500) or anti-SKAP-Nter antibody (1:2000).

### Spermatocyte squashes, nuclei spreads and immunostaining

Spermatocyte squashes were obtained as described previously (Page *et al*. 1998, Viera *et al*. 2002). After fixation, squashed spermatocytes were rinsed three times in PBS for 5 min, and incubated with mouse anti-SYCP3 (1:100), ACA/CREST serum (1:30) and guinea pig anti-SKAP (1:100) antibodies diluted in PBS at 4 °C overnight. Nuclei spreads were prepared according to the drying-down technique as described in Peters *et al*. (1997) and immunostained as described in Grey *et al*. (2009). For studies in HeLa cells, cells were grown on coverslips and transfected with GFP-SKAP overnight. For immunofluorescence, HeLa cells were washed with PBS, fixed in methanol for 10 min and permeabilized in PBS/0.05% Triton X-100/5% fetal veal serum for 1 h. Cells were incubated with primary antibodies (rabbit anti-GFP, 1:1000 and mouse anti-tubulin clone DM1a, 1:500) in blocking solution at room temperature (RT) for 1 h. After washes in PBS containing 0.1% Tween-20 (PBS-T), cells were incubated with secondary antibodies for 1 h at RT and then stained with DAPI (1:2000). Images were recorded with a Zeiss Axioimager Z1 microscope equipped with a Coolsnap HQ2 camera at 1×1 binning with a 100×, 1.3 NA Olympus U-PlanApo objective. Images were imported into Image J for further processing.

### Sperm count in adult testes

Epididymes of adult testes were isolated and mechanically homogenized in 200 μl PBS and then transferred to Eppendorf tubes containing 800 μl of PBS. Samples were then diluted at 1:50 and sperm cells counted with a hemocytometer.

### Paraffin-embedded tissue sections, immunostaining and TUNEL assay

Testes were fixed in Bouin's solution for hematoxylin–eosin (HE) or periodic acid–Schiff (PAS) staining for 5 h (juvenile testes) or overnight at RT, or in 4% paraformaldehyde (for immunostaining) at RT. Testes were then embedded in paraffin and cut in 3 μm slices. HE- and PAS-stained sections were scanned using the automated tissue slide scanning tool of a Hamamatsu NanoZoomer Digital Pathology system. TUNEL assays were performed with the DeadEnd Fluorometric TUNEL System (Promega), according to the manufacturer's protocol. For immunostaining, sections were deparaffinized, washed and permeabilized in 0.1% Triton X-100/PBS. Sections were then blocked in blocking buffer (10% donkey serum, 0.05% Tween-20, 0.05% Triton X-100 in PBS) for 30 min at RT and incubated with the primary antibodies (anti-SKAP, 1:100; anti-N-ter SKAP, 1:2000; anti-TRA98, 1:100 and anti-SOX9, 1:100 in blocking buffer) at RT overnight. Secondary antibodies were also diluted in blocking buffer and added at 37 °C for 1 h. Images were acquired with a sCMOS ORCA Flash 4.2 camera (Hamamatsu), an Axioimager Z2 microscope (Carl Zeiss), a 63× PL APO 1.4 lens, DAPI (FS49), FITC (FS38 HE), Texas Red (FS45), Cy3 (FS43) and Cy5 (FS50) fluorescence filter sets.

### Oocyte collection, culture and microinjection

Oocytes were collected from 11-week-old OF1 mice and from 11- to 16-week-old WT and SKAP-deficient females, as described previously (Verlhac *et al*. 1994), and maintained in prophase I arrest in M2+BSA medium supplemented with 1 μM milrinone (Reis *et al*. 2006). At this stage, they were microinjected with cRNAs encoding Histone-RFP using an Eppendorf Femtojet microinjector. Oocytes were kept in prophase I for 1–2 h to allow cRNA expression and then released in M2+BSA without milrinone to trigger meiosis resumption. Cultures and live imaging acquisition were carried out under oil at 37 °C.

### In vitro transcription of cRNAs for oocyte microinjection

The pRN3-Histone-GFP construct (Dumont *et al*. 2007) was used to label chromosomes and follow them during anaphase I. *In vitro* synthesis of capped cRNAs was performed as described previously (Dumont *et al*. 2007). cRNAs were centrifuged at 13 500 ***g*** at 4 °C for 45 min before microinjection.

### Live imaging of oocytes

Spinning disk images of live oocytes in M2+BSA medium were acquired at 37 °C using a Plan-APO 40×/1.25 NA objective on a Leica DMI6000B microscope enclosed in a thermostatic chamber (Life Imaging Service, Basel, Switzerland) equipped with a CoolSnap HQ2/CCD-camera (Princeton Instruments) coupled to a Sutter filter wheel (Roper Scientific, Munich, Germany) and a Yokogawa CSU-X1-M1 spinning disk. The Metamorph software (Universal Imaging, Bedford Hills, New York, USA) was used for data collection. For live chromosome imaging, seven *z*-planes were acquired, spaced 3 μm, every 30 min starting 0530 h after nuclear envelope breakdown, with a power of 10% of laser −581 nm and 200 ms acquisition time.

### Immunofluorescence staining of oocytes

Oocytes were fixed on coated coverslips with paraformaldehyde or glutaraldehyde, as described previously (Verlhac *et al*. 1994, Terret *et al*. 2003). After incubation with the anti-SKAP (1:200) or rat monoclonal anti-tyrosinated α-tubulin antibody (YL1/2, 1:100, Abcam) and washes, oocytes were incubated with anti-guinea pig-Cy3 or anti-rat-Cy3 secondary antibodies respectively (Jackson ImmunoResearch Laboratories, Inc.). Coverslips were mounted in Prolong Gold with DAPI (Life Technologies). Image acquisition of fixed oocytes was performed using the SP5/AOBS confocal microscope equipped with a Plan APO 63×/1.4 N.A. objective.

### Oocyte image analysis

3D analysis of spindle morphology, spindle length and chromosome volume in metaphase II oocytes was performed using Imaris (Bitplane, Zurich, Switzerland).

## Results

### SKAP is specifically expressed in the germline

**Figure 1:**
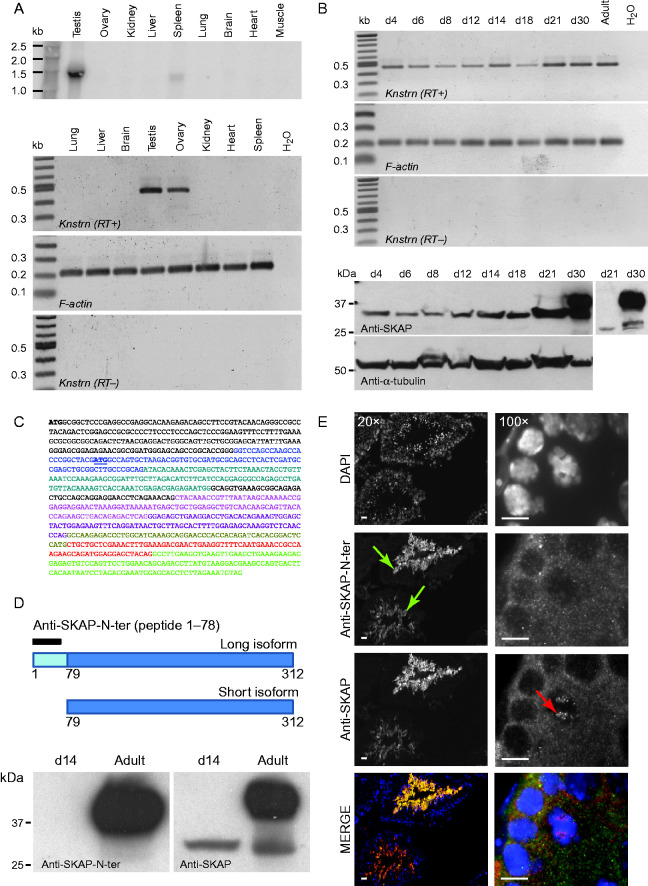
SKAP germline-specific expression. (A) Northern blot (upper panel) and RT-PCR (lower panels) analysis of RNA from various tissues of adult mice. (B) RT-PCR (upper panels) and western blot (lower panels) analysis of SKAP expression in juvenile (day 4–30ppt) and adult mouse testes. Insert in the lower panel: day 21 and 30ppt lanes after shorter exposure. F-actin and anti-α tubulin were used as controls for RT-PCR and western blotting respectively. (C) cDNA sequence of *Knstrn*, the gene encoding SKAP (NM_026412.3). The alternative in-frame start codon (in blue and underlined) in the second exon encodes a protein that is 9 kDa shorter than full-length SKAP. Different colors represent different exons. (D) Top: cartoon representing the two SKAP isoforms. The region upstream of the alternative start codon is in light blue. The black bar indicates the peptide used to produce the anti-SKAP-N-ter antibody. Bottom: western blotting of juvenile (day 14ppt) and adult testis extracts with the anti-SKAP-N-ter antibody (left panel) and the anti-SKAP antibody. (E) Localization of the long (anti-SKAP-N-ter antibody) and short (anti-SKAP antibody) SKAP isoforms in adult testes. Left panels: seminiferous tubules (20× magnification). Right panels: cells at the periphery of a seminiferous tubule (100× magnification). Green arrows, SKAP-positive spermatids; red arrow, SKAP-positive spermatogonia. Scale bar: 10 μm.

To evaluate a germ cell-specific function of mouse SKAP, we determined its expression profile in various adult tissues (Fig. 1A). By northern blot, we detected a transcript of about 1.45 kb in the testis and a slightly shorter low-abundance transcript in the spleen. No transcript was detected in other tissues in the particular ovary. The size of the testis transcript is consistent with the referenced size of *Knstrn* mRNA, which is 1.5 kb (AK006328.1). By RT-PCR, using primers designed to amplify cDNA from exon 2 to exon 7, we detected a transcript in the adult testis and ovary but not in the spleen (Fig. 1A). The detection of *Knstrn* by RT-PCR in the ovary might reflect the higher sensitivity of this assay. We concluded that mouse SKAP was specifically expressed in male and female gonads.

In the male mouse, the germline was composed of different cell types representing successive stages of maturation, from spermatogonia to spermatozoa. Undifferentiated spermatogonia divide mitotically before they enter meiotic S-phase followed by the two meiotic divisions (MI and MII). Whereas MII strongly resembles mitosis, where chromosomes are bi-oriented and sister chromatids are pulled to opposite poles, MI displays mono-oriented chromosomes that organize the spindles in such a way that both sister chromatids of a given homolog are pulled towards the same pole. In order to determine whether SKAP is present during one, if not all, of these steps, we determined its expression kinetics before and during the first wave of meiosis in juvenile testes. In the mouse, spermatogonia enter meiosis at about day 8 post-partum (ppt) almost synchronously and progress through prophase I until they reach metaphase I around day 21ppt and metaphase II around day 22ppt. Accordingly, we monitored the presence of the *SKAP* transcript and protein in testes from day 4ppt to day 30ppt (Fig. 1B). The transcript and the protein were readily detectable before entry into meiosis (day 4ppt), and maintained at all monitored ages. By western blot, we detected a signal at two molecular weights: one at 30kDa, present at all ages, and a larger protein of 40 kDa which was only detected in adult testes, where it represented the major form of SKAP. Both proteins were absent in mice deficient for SKAP (see below). The occurrence of different protein sizes can be explained either by alternatively spliced transcripts, by the usage of alternative start codons or by post-transcriptional modifications. In the case of *Knstrn*, an alternative transcript (alternative version of *exon 8*) was indeed referenced in databases (NCBI, XM006499894.1, transcript variant *X1*). *Knstrn X1* would be translated into a protein of a size compatible with the smaller protein. Alternatively, *Knstrn* mRNA also possesses an in-frame start codon at the beginning of *exon 2* (Fig. 1C), which could also give rise to a protein with the size of the smaller SKAP. To distinguish between these possibilities, we took advantage of an N-terminal specific anti-SKAP antibody generated by I.M. Cheeseman's laboratory, kindly provided before its publication. The antibody was raised against a peptide of the N-terminal part of the protein (amino acids 1–78), which is located upstream of the alternative start codon (Fig. 1D). If the smaller SKAP isoform arose from the alternative start codon in *exon 2* rather than from the alternative *X1* transcript, the anti-SKAP N-ter antibody was predicted to recognize only the larger isoform of SKAP. This was the case: whereas with the anti-SKAP N-ter antibody, the larger isoform was still detected in adult testes, the smaller isoform in either juvenile or adult testes remained undetectable (Fig. 1D). Furthermore, staining of SKAP in testis sections using full-length and N-ter specific antibodies revealed two distinct and specific localization patterns; in adult testis sections, both antibodies displayed a very strong signal in the cytoplasm of elongating spermatids (Fig. 1E, left panels, green arrows, Figure S1, see section on supplementary data given at the end of this article), but only the full-length anti-SKAP antibody revealed a nuclear signal close to the periphery of the seminiferous tubules, where spermatogonia and meiotic cells localize (Fig. 1E, right panel, red arrow). These observations led us to conclude that SKAP exists in two distinct forms: a 40 kDa isoform present only in the cytoplasm of maturating spermatids and a 30 kDa isoform present in the nucleus of earlier-stage germ cells. In this work, we focused on the smaller SKAP isoform, present in spermatocytes and spermatogonia of juvenile and adult mice.

### SKAP localizes at the kinetochores from metaphase to telophase in mitotic and meiotic germ cells

**Figure 2:**
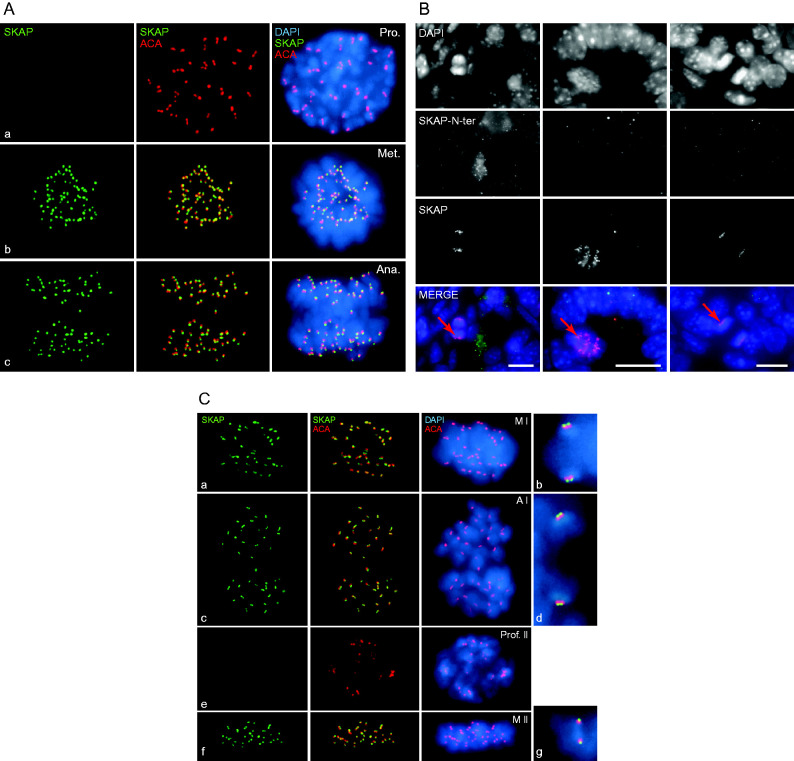
The smaller SKAP isoform localizes at the kinetochores and at the central spindle of metaphase to telophase spermatogonia and spermatocytes. (A, C) Squashed nuclei stained with the anti-SKAP antibody (green), ACA/CREST serum (centromeres; red) and DAPI (blue). (A) Spermatogonia in (a) prophase, (b) metaphase and (c) anaphase. (B) Sections of day 7ppt testes stained with the anti-SKAP and anti-SKAP-N-ter antibodies. Red arrows indicate SKAP localization at the kinetochores of anaphase to telophase spermatogonia. The dotted signal detected with the anti-SKAP-N-ter antibody (left panel) did not co-localize with DAPI and therefore was probably non-specific. Scale bars: 10 μm. (C) Spermatocytes in (a) metaphase I, (c) anaphase I, (e) prophase II, (f) metaphase II and (b, d and g) are higher magnifications of the left panels.

**Figure 3:**
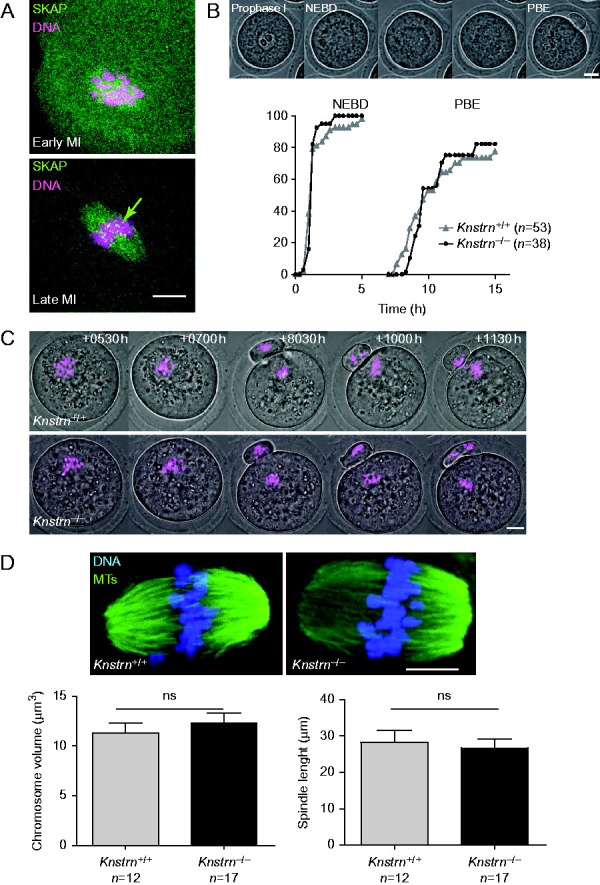
SKAP localizes at the kinetochores in female germ cells, but its absence has no severe impact on oocyte maturation. (A) SKAP is expressed at the kinetochores at metaphase I during meiosis I (MI). Oocytes were collected and fixed 1 h (upper panel) and 7 h after nuclear envelope breakdown (NEBD) (lower panel). SKAP expression was assessed using the anti-SKAP antibody (green). Chromosomes are highlighted in magenta. SKAP accumulates at the kinetochores at metaphase I (green arrow). (B) SKAP is not required for timely progression into meiosis I. Top: oocytes were followed by live imaging during meiotic maturation (NEBD) and the first polar body extrusion (PBE). Bottom: *Knstrn*^*+/+*^ and *Knstrn*^−/−^ oocytes resumed meiosis and extruded the first polar body with similar kinetics. (C) SKAP is not required for correct segregation of bivalents in anaphase I. Oocytes expressing Histone-RFP (magenta) were followed starting at 0530 h after NEBD. Anaphase I progression was comparable in WT (*n*=10) and *Knstrn*^−/−^ (*n*=13) oocytes (two independent experiments). The percentage of oocytes with lagging chromosomes at anaphase I was comparable in the two groups (20% for controls and 8% for *Knstrn*^−/−^ oocytes). A maximal Z-projection of all planes is presented for Histone-RFP (magenta) labeling. Time (in h: min) after NEBD is shown. (D) SKAP is not required for metaphase II spindle assembly and sister chromatid alignment in the metaphase plate. *Knstrn*^−/−^ oocytes display canonical metaphase II spindles with well-aligned chromosomes on the metaphase plate, consistent with proper progression into anaphase I. Spindle size and chromosome volume are comparable in *Knstrn*^*+/+*^ and *Knstrn*^−/−^ oocytes (lower histograms). Metaphase II oocytes were fixed 16 h after NEBD. Green, microtubules; blue, chromosomes. All scale bars are 10 μm.

As we detected the small isoform of SKAP at the periphery of seminiferous tubules, a position which is occupied by spermatogonia and early spermatocytes, we examined its signal more closely in the spermatogonia of squashed seminiferous tubules of adult testes (Fig. 2A). In those cells, SKAP localized at the kinetochores. We further confirmed this localization on day 7ppt testis sections where only spermatogonia were present. In those sections, SKAP localized at the kinetochores and the central spindle of metaphase to telophase spermatogonia (Fig. 2B). Interestingly, SKAP was also detected at the kinetochores in spermatocytes in metaphase I, anaphase I and metaphase II spermatocytes (Figs 2C and S1A). Moreover, SKAP localized externally of the inner kinetochore signals revealed by the ACA-serum (Fig. 2C) or the SYCP3 signals, located at the inner centromere domains (Figure S2A, see section on supplementary data given at the end of this article) (Parra *et al*. 2004). During spermatogonial mitotic prophase (Fig. 2B) or meiotic prophase however, SKAP remained undetectable (Figs 2C and S1B). In females, we also detected SKAP at the kinetochores of metaphase I oocytes (Fig. 3A).

These observations support the idea for a role of SKAP at the interface of kinetochores and microtubules as already suggested for immortalized cells (Schmidt *et al*. 2010, Huang *et al*. 2012). Indeed, mouse SKAP seems to behave the same way as its human counterpart since when ectopically expressed in HeLa cells, it also localizes at the kinetochores during the prometaphase, at the kinetochores and spindles during the metaphase and at the midzone during the anaphase such as hSKAP (Figure S2C).

### Fertility is reduced in SKAP-deficient mice

**Figure 4:**
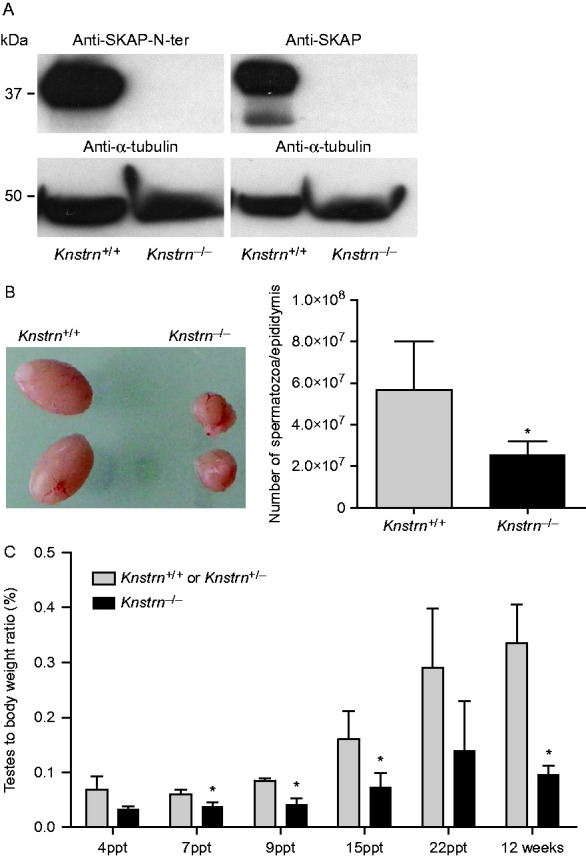
SKAP-deficient mice have smaller testes and produce less sperm. (A) Western blot analysis of WT and SKAP-deficient adult testis extracts. α-Tubulin was used as the loading control. (B) Representative images of *Knstrn*^*+/+*^ and *Knstrn*^−/−^ adult testes and histogram showing the mean number of sperm cells ±s.d./epididymis in adult (15-week-old) WT and mutant testes (*P*=0.013, *t*-test, *n*=4 testes). (C) Testis weight is significantly lower in SKAP-deficient compared with WT mice. Body and testes were weighed in *Knstrn*^*+/+*^*, Knstrn*^+/−^ and *Knstrn*^−/−^ mice from day 4ppt up to 12 weeks of age. From day 7ppt, the testes-to-body weight ratio of *Knstrn*^−/−^ mice was significantly lower than that in WT animals (*P<0.05, at day 22 *P*=0.08, Fisher's exact test; *n*=6 animals).

The expression and localization profile of SKAP in germ cells suggested a role during gametogenesis. To unravel its role in this process, we generated a mouse line where exon 3 of *Knstrn* was deleted, creating a frameshift and an early stop codon, that should have led to the production of a truncated protein of 146 amino acids and a mobility of about 16 kDa. However, we could not detect any SKAP protein in testis extracts of adult *Knstn*^−/−^ mice (Fig. 4A). Conversely, both isoforms were normally expressed in *Knstrn*^*+/*−^ mice (data not shown).

*Knstrn*^−/−^ mice grew normally without any obvious developmental defects and had lifespans similar to that of WT mice. This indicates that *in vivo* SKAP is not essential for viability, differently from *in vitro*RNAi studies performed on HeLa cells. Intercrosses between heterozygous males and females resulted in offspring with an expected Mendelian ratio (43 *Knstrn*^+/+^:89 *Knstrn*^+/−^:32 *Knstrn*^−/−^, *P*=0.263, *χ*^2^-test). Intercrosses involving one or both heterozygous parents led to WT litter sizes. In contrast, even though SKAP-deficient male and female mice were fertile, intercrosses involving one homozygous *Knstrn*^−/−^ parent led to reduced litter size: 4.1±2.5 pups with a *Knstrn*^−/−^ mother and 4.8±1.9 pups with a *Knstrn*^−/−^ father compared with 6.9±1.88 pups in crosses between heterozygous animals (*P*<0.0011 and 0.039 respectively, *t*-test) (Supplementary Table ST1, see section on supplementary data given at the end of this articles). To investigate the reduction in litter size in *Knstrn*^−/−^ female mice, we analyzed histological ovary sections of juvenile (day 4ppt) and adult female mice at various ages (2, 6 and 11 months). At all assessed ages, ovaries were of normal size and contained only a slight, statistically non-significant reduction of the number of primordial, primary and advanced follicles (data not shown). We thus suspected a defect during the meiotic divisions, but SKAP deficiency did not have a severe impact on oocyte maturation. Timely progression into meiosis I was not affected by the absence of SKAP. Oocytes resume meiosis I normally and extrude their first polar bodies with frequencies and kinetics comparable to WT controls (Fig. 3B). Furthermore, by following chromosome segregation by live imaging, we did not detect any obvious defects in either of the two meiotic divisions (Fig. 3C). Consistent with this, metaphase II oocytes did not present any obvious defects in spindle morphology (Fig. 3D). In addition, the length of meiotic spindles and the volume occupied by chromosomes were not affected in *Knstrn*^−/−^ metaphase II oocytes (Fig. 3D).

**Figure 5:**
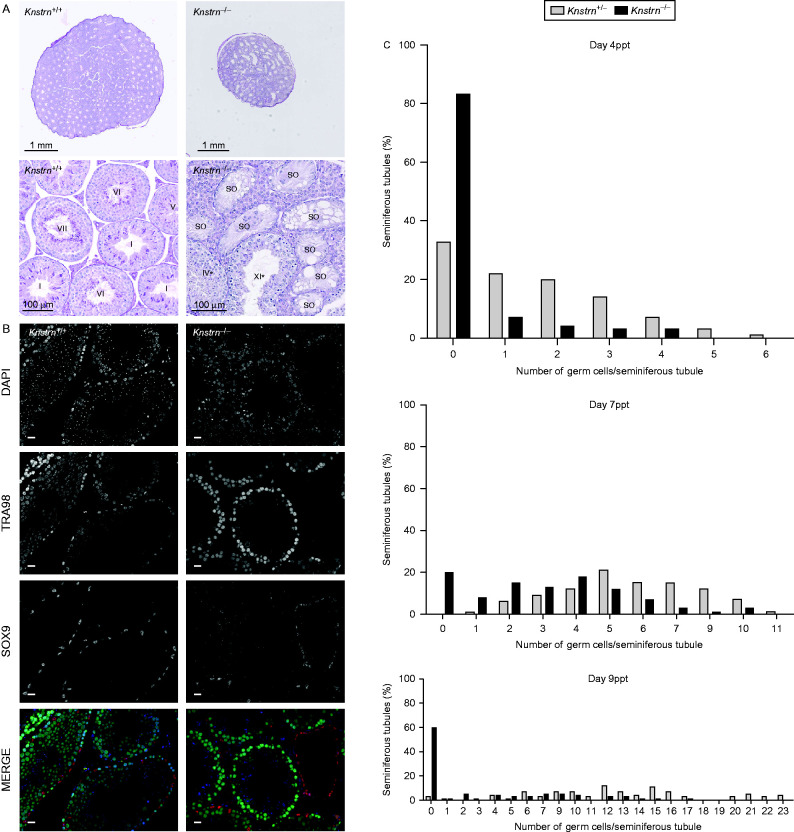
Seminiferous tubules of SKAP-deficient mice are partially or completely devoid of germ cells due to a high number of tubules devoid of germ cells. (A) Cross-sections of WT and *Knstrn*^−/−^ adult testes stained with PAS. Middle and lower panels: higher magnification of individual seminiferous tubules. Roman numbers indicate the developmental stage of each tubule. SO: Sertoli only; *: presence also of germ cells that are not at the expected developmental stage; arrows: Sertoli cell vacuolization (B) Immunofluorescence analysis of cross-sections from *Knstrn*^*+/+*^ and *Knstrn*^−/−^ adult testes. Nuclei were stained with DAPI (blue in the lower panel), germ cells with an anti-TRA98 (green in the lower panel) and Sertoli cells with an anti-SOX9 antibody (red in the lower panel). Scale bar: 10 μm. (C) Histograms represent the percentage of *Knstrn*^+/−^ (gray) and *Knstrn*^−/−^ (black) seminiferous tubules that contain 0–6 (day 4ppt testes), 0–10 (day 7ppt testes) or 0–21 (day 9ppt testes) germ cells. For statistics, see Table 3.

Conversely, in SKAP-deficient males, testis size and sperm production were significantly reduced compared with WT littermates (Fig. 4B). The analysis of PAS-stained histological sections of adult testes revealed a rather complex phenotype; although mutant germ cells did undergo all stages of spermatogenesis, as evidenced by the presence of all cell types derived from the germ cell lineage (spermatogonia, spermatocytes, spermatids and spermatozoa), less than 4% (3.14±2.3%, *n*=4 mice) of seminiferous tubules had a normal phenotype. Most of the tubules showed signs of Sertoli cell vacuolization, which is generally caused by severe germ cell depletion through Sertoli cell endocytosis. Indeed, tubules with vacuolization contained fewer germ cells and the architecture of the remaining cells was disorganized (Fig. 5A). Moreover, immunostaining analysis of germ cells (anti-TRA98) and Sertoli cells (anti-SOX9) confirmed that tubules were almost completely devoid of germ cells, with only a single row of Sertoli cells and some spermatogonia at the basal lamina were frequent (Fig. 5B).

### Spermatogenesis is disrupted shortly after birth

To define the onset of the heterogeneous disruption of spermatogenesis in *Knstrn*^−/−^ mice, we analyzed testis sections of juvenile WT and mutant mice. During the first wave of spermatogenesis, germ cells almost synchronously enter meiosis; this allowed us to timely dissect germ cell differentiation in a series of successive events defined as follows: self-renewal (days 0–4ppt); proliferation and differentiation (days 4–7ppt); meiosis (days 8–21ppt); and spermiogenesis (days 21–35ppt) (Meistrich 1993, de Rooij & Russell 2000, McLaren 2000, de Rooij 2001, Drumond *et al*. 2011). We examined the testis-to-body weight ratio of mutant and WT mice at ages, representative for these events (days 4, 7, 9, 15, 22ppt and adult). As early as day 7ppt, the testis-to-body weight ratio was significantly different in *Knstrn*^−/−^ compared with *Knstrn*^+/−^ mice (0.061±0.008 vs 0.038±0.007, respectively, *P*<0.05, *t*-test) and mutant testes stayed significantly smaller even in adult mice (Fig. 4C).

To determine the cause of this reduction, we quantified the number of germ cells (anti-TRA98) and Sertoli cells (anti-SOX9) in seminiferous tubules of WT and *Knstrn*^−/−^ mice at days 4, 7 and 9ppt. The number of germ cells but not Sertoli cells in *Knstrn*^−/−^ seminiferous tubules was significantly reduced at all assessed time points compared with controls (*P*<0.0001, Mann–Whitney test) (Table 1)

### Apoptosis in SKAP-deficient testes occurs within a specific time window

To determine whether *Knstrn*^−/−^ mice present enhanced apoptosis, we investigated apoptosis by performing a TUNEL assay in adult and day 4 and 9ppt testes. Apoptosis was significantly enhanced at day 9ppt, but not at day 4ppt nor in adult *Knstrn*^−/−^ testes (Table 2 and Figure S3, see section on supplementary data given at the end of this article).

Closer examination of *Knstrn*^−/−^ testis sections showed that the reduction of the total number of germ cells per tubule was partially caused by the high percentage of tubule sections containing no germ cells at all (Fig. 5C). At day 4ppt, this was most likely due to reduced proliferation of self-renewing spermatogonia, whereas at day 9ppt, enhanced apoptosis could be the explanation (Table 2). Interestingly, in day 7ppt *Knstrn*^−/−^ testes, germ cells were present in 80% of all the observed tubule sections (Fig. 5C and Table 3). However, *Knstrn*^−/−^ germ cells (spermatogonia) did not proliferate like WT cells because their total number per tubule was still significantly lower than in the wild type (3.1±1.95 in *Knstrn*^−/−^ vs 5,6±2.06 in *Knstrn*^+/−^, *P*<0.001, Mann–Whitney, *n*: at least 99 tubules and 4 testes).

## Discussion

SKAP was first discovered in immortalized cell lines as an interactor of kinetochore- and microtubule-associated proteins such as SPAG5 (ASTRIN) and CENPE, revealing a role for SKAP in stabilizing the interaction between microtubules and kinetochores. In human and chicken transformed cell lines, knocking down SKAP has deleterious effects on the mitotic success rate (Schmidt *et al*. 2010, Dunsch *et al*. 2011, Huang *et al*. 2012). Here we found that in the mouse, SKAP is dispensable for viability but essential for normal fertility, its expression being restricted to the germline. We showed that in testes, SKAP exists in two distinct isoforms: a 40 kDa isoform, present in the cytoplasm of maturing spermatids, and a 30 kDa isoform lacking the first 78 of the protein, present at the outer kinetochores of spermatogonia, spermatocytes and oocytes. The first 78 amino acids may play a role in addressing the larger isoform to the cytoplasm of elongating spermatids, but we did not identify any known protein domains in the N-terminal part of SKAP, and all previously described protein interaction domains, such as those interacting with SPAG5 and CENPE, are located downstream of the alternative start codon and, hence, also present in the smaller isoform (Dunsch *et al*. 2011, Huang *et al*. 2012). As the two isoforms localize very distinctly, they could have distinct roles during spermatogenesis. Whereas spermatogonia and spermatocytes undergo mitotic and meiotic divisions and thus build kinetochores, elongating spermatids are non-dividing haploid cells that go through strong DNA compaction, nuclear elongation, organelle reorganization and progressive loss of their cytoplasm. In spermatids, kinetochores do not form, but microtubules play an essential role in the reorganization and the elongation of their nuclei (Moreno & Schatten 2000). The larger isoform of SKAP might play a role in these processes. However, in the absence of SKAP, spermatogenesis seems to be affected at a very early stage, even before entry in meiosis, suggesting that the spermatogenesis defect of SKAP-deficient mice is mainly due to the absence of the small isoform. In *Knstrn*^−/−^ mice, we observed a reduced germ cell-to-Sertoli cell ratio as early as day 4ppt and an enhanced apoptosis at day 9ppt but not in day 4ppt and adult testes. Enhanced apoptosis in days 9–10ppt testes is reminiscent of mutants that present dysregulation of spermatogonial differentiation and/or proliferation such as KIT/SCR, GDNF, GFRA1 and RET (Blume-Jensen *et al*. 2000; Naughton *et al*. 2006). For instance, in mice with reduced Kit/SCF-R activity, extensive apoptosis is observed in day 10ppt testes but not in adult testes. In those mutants, differentiated germ cells are completely absent and seminiferous tubules contain only Sertoli cells and undifferentiated spermatogonia (Naughton *et al*. 2006). Conversely, in SKAP-deficient mice, spermatogonial differentiation did not seem to be affected because some tubules contained all cell types produced during spermatogenesis. Reduced spermatogonial proliferation could be an alternative explanation for the strong decrease in the number of germ cells in some SKAP-deficient seminiferous tubules. Indeed, during spermatogenesis, spermatogonia go through eight or nine divisions, on average, before differentiating into spermatocytes (de Rooij & Lok 1983, Tegelenbosch & de Rooij 1993), whereas spermatocytes only go through two successive meiotic divisions. Therefore, in theory, one spermatogonium is capable of rendering up to 4096 spermatozoa (Russell *et al*. 1990), hence the regulation of the number of germ cells in a seminiferous tubule is already determined by the pool of self-renewing and mitotic spermatogonia. This regulation also ensures the appropriate germ cell-to-Sertoli cell ratio (de Rooij & Janssen 1987, de Rooij & Lok 1987). Tubules that fail to produce a sufficient number of differentiated spermatogonia and thus an equilibrated germ cell-to-Sertoli cell ratio fail to proceed through spermatogenesis and eventually end up showing a ‘Sertoli-only’ phenotype with strong cell vacuolization (Raverot *et al*. 2005, Oatley *et al*. 2006). SKAP deficiency could variably affect the mitotic rate of self-renewing and proliferating spermatogonia and lead to different phenotypic outcomes: seminiferous tubules in which differentiating spermatogonia have never been generated (‘Sertoli-only’); seminiferous tubules in which spermatogonia have been generated, but divided at insufficient rates, creating an imbalanced germ cell-to-Sertoli cell ratio leading to apoptosis (‘Sertoli-only’ tubules and tubules with some remaining germ cells); and seminiferous tubules in which a sufficient amount of healthy spermatogonia could eventually enter meiosis and produce, at least in part, functional sperm. This implicates that SKAP is not completely indispensable for the completion of mitosis and meiosis. Similarly, in humans and chickens, after an initial block in the metaphase, SKAP-deficient cells eventually resume mitosis (Fang *et al*. 2009, Schmidt *et al*. 2010). In these cells, SKAP stabilizes bi-oriented kinetochore–spindle attachments and the observed mitotic arrest is possibly due to SAC activation, caused by the reduction of inter-kinetochore tension, leading to the inability to maintain chromosome alignments (Fang *et al*. 2009, Schmidt *et al*. 2010). A similar function for SKAP during mouse germ cell mitosis could explain why the frequently dividing spermatogonia were the most severely affected germ cell population in *Knstrn*^−/−^ mice. Spermatogonia, which, in spite of the absence of SKAP, survived several rounds of mitosis and thus entered meiosis, only underwent two more ‘chances’ to missegregate their chromosomes before resuming spermatogenesis and thus produced functional sperm. A kinetochore–microtubule stabilization function of SKAP might also explain the less severe phenotype in female germ cells as fewer cell divisions are involved in oocyte differentiation and as the spindle is organized differently in females with an acentrosomal assembly (Howe & FitzHarris 2013). In oocytes, defects in chromosome segregation at anaphase I usually have an impact on metaphase II spindle organization (Terret *et al*. 2003). This was not the case in SKAP-deficient oocytes. Thus, even though our results strongly argue against a major role for SKAP in meiosis I progression or in metaphase II arrest in females, we cannot exclude a minor contribution of SKAP to chromosome segregation, not detected by the techniques we used. It is puzzling that normal amounts of apparently healthy oocytes lead to lower litter sizes in crosses involving female SKAP-deficient mice. It might be interesting to assess the *in utero* development of embryos from SKAP-deficient mothers in order to determine at what stage of the embryonic development, the litter size decreases.

Taken together, our work leads us to conclude that SKAP is a germline-specific protein that in testes exists in two isoforms: a small isoform, only present during early stages of spermatogenesis, at the outer kinetochores of spermatogonia and spermatocytes, and a larger isoform, only detectable in late-stage elongating spermatids. SKAP deficiency in the mouse results in a spermatogenesis defect without completely abolishing the formation of mature spermatozoa. The observed phenotype is mainly due to a malfunction in early spermatogenesis, presumably during the mitotic divisions of spermatogonia before entering meiosis. Even though the phenotype seems less severe of what has been observed in human immortalized hSKAP knockdown cells, our results support the idea of a role of SKAP in the stabilization of the interaction of microtubules with the outer kinetochores during mitosis. The gametogenesis defects that we detected in *Knstrn*^−/−^ mice suggest that *KNSTRN* is an interesting candidate gene to be screened in infertile or hypo-fertile patients.

## Supplementary data

This is linked to the online version of the paper at http://dx.doi.org/10.1530/REP-15-0451.

## Author contribution statement

C Grey developed concepts and approaches, performed the experiments and prepared the manuscript; J Espeut performed the experiments, developed concepts and revised the manuscript; R Ametsitsi performed the experiments, B de Massy raised funding, developed concepts and revised the manuscript; R Ametsitsi, R Kumar, M Luksza, M-H Verhlac and J A Suja performed the experiments.

## Figures and Tables

**Table 1:** Cell count in seminiferous tubules of WT and SKAP-deficient mouse testes at days 4, 7 and 9ppt. The number of germ cells in SKAP-deficient mice is reduced already early after birth. After immunostaining with an anti-TRA98 antibody (germ cells) and an anti-SOX9 antibody (Sertoli cells), cells were counted in cross-sections of day 4ppt, day 7ppt and day 9ppt WT and knockout (KO) testes. The total amount of cells per tubule was monitored by DAPI staining

	**WT 4dpp**	**KO 4dpp**	***P***	**WT 7dpp**	**KO 7dpp**	***P***	**WT 9dpp**	**KO 9dpp**	***P***
Total cells/tubule	21.76	22.14	0.624	26.57	23.20	<0.0001	43.70	34.72	<0.0001
Sertoli cells	20.21	21.26	0.0969	20.99	20.08	0.131	31	31.16	0.48
Germ cells	1.5	0.34	<0.0001	5.58	3.07	<0.0001	12.6	3.56	<0.0001
N tubules	100	100		100	100		75	75	
N testes	2	2		4	4		4	4	

The number of germ cells was significantly reduced in SKAP-deficient mice at all ages compared with WT controls (*P*<0.0001, Mann–Whitney non-parametric test), whereas the number of Sertoli cells was not different in the two groups.

**Table 2:** Assessment of apoptosis in seminiferous tubules of WT and SKAP-deficient testes in adult and day 4 and 9ppt mice. Apoptosis in testes is significantly higher in SKAP-deficient mice than in WT mice at day 9ppt, but not at day 4ppt or in adults. Apoptotic cells in the seminiferous tubules of WT and knockout (KO) testes were detected by TUNEL assay. Seminiferous tubules containing two or more apoptotic cells were counted as apoptosis-positive

	**WT 4dpp**	**KO 4dpp**	**WT 9dpp**	**KO 9dpp**	**WT adult**	**KO adult**
≥2 apoptotic cells per tubule	1.4	3.4	17.6	36.4	28.0	30.5
Total number of tubules counted	300	300	300	300	201	201
Number of testes used	4	4	4	4	2	4
*P* (Fisher's exact test	0.17		0.0006		0.8	

The number of apoptosis-positive tubules was significantly higher in KO than in WT mice only at day 9ppt (*P*=0.0006, Fisher's exact test).

**Table 3:** Germ cell per tubule count of WT and SKAP-deficient mouse testes at days 4, 7 and 9ppt. The percentage of seminiferous tubules devoid of germ cells is highest in day 4ppt and day 9ppt *Knstrn*^−/−^ testes. Percentage of tubules that have at least one or no germ cells (*n*=75 tubules per genotype and age)

**Germ cells/tubule**	***Knstrn*** ^**+/−**^	***Knstrn*** ^**−/−**^	***P*** **(Fisher's exact test)**
Day 4ppt nb with ≥1	70	17	
Day 4ppt nb with 0	35	88	<0.0001
Day 7ppt nb with ≥1	99	80	
Day 7ppt nb with 0	0	20	<0.0001
Day 9ppt nb with ≥1	73	30	
Day 9ppt nb with 0	2	45	<0.0001
